# A new species of the ant genus *Leptogenys* Roger, 1861 (Hymenoptera: Formicidae) from India

**DOI:** 10.3897/BDJ.6.e25016

**Published:** 2018-07-12

**Authors:** Aijaz Ahmad Wachkoo, Amir Maqbool, Shahid Ali Akbar, Mostafa Rezk Sharaf

**Affiliations:** 1 Government Degree College, Shopian, Jammu & Kashmir, India; 2 Government College for Women, M.A. Road, Srinagar, Jammu & Kashmir, India; 3 Central Institute of Temperate Horticulture, Srinagar, Jammu & Kashmir, India; 4 King Saud University, Riyadh, Saudi Arabia

**Keywords:** *
Leptogenys
*, *chinensis*-group, Ponerinae, Western Himalayas, new species

## Abstract

**Background:**

There are no well defined *Leptogenys* species groups based on the worker morphology from the Oriental region Arimoto (2017). *Leptogenys chinensis* forms a complex species group with closely related species having little morphological changes Wilson (1958), Sarnat and Economo (2012). From the Oriental region, there are currently 9 species belonging to the *L. chinensis* group. The group is diagnosed by having edentate masticatory margin of the mandible, smooth body surface, elongate antennae and metallic green cuticle. The species included are: *L. assamensis*; *L. chinensis*; *L. confucii*; *L. kraepelini*; *L. laeviterga*; *L. pangui*; *L. peuqueti*; *L. stenocheilos* and *L. sunzii.*

**New information:**

*Leptogenys bhartii* sp. n., a new ponerine ant species from Western Himalayas, India, is described and illustrated based on the worker caste. The new species belongs to the *Leptogenys chinensis* group and mostly resembles *Leptogenys chinensis* (Mayr, 1870). In the *L. chinensis* group, the original description of *L. stenocheilos* is insufficient as it lacks information about type material. As there is no further detailing of this species in the available literature, it is difficult to ascertain its valid status Xu and He (2015) and is therefore, considered a *species inquirenda* herewith. A revised key to the known species of *chinensis*-group in the Oriental Region is provided.

## Introduction

The ant genus *Leptogenys* Roger, 1861 is globally represented by 335 described species and subspecies (AntCat 2018). The genus is currently represented by about 100 species in the Oriental Region (Xu and He 2015, Zyranin 2016). As anticipated by Bharti and Wachkoo (2013), nearly half of the Indian *Leptogenys* await discovery, however, 35 species and subspecies of the genus are reported from the country (Bharti and Wachkoo 2013, AntCat 2018). The ants of this genus are known to have ergatoid queens and gamergate reproductives and are considered an ideal taxon for the study of the evolution of reproductive behaviour in ants (Peters 2012, Bharti and Wachkoo 2013, Schmidt and Shattuck 2014). Global taxonomic revision still eludes the genus, however; some of the recent contributions to the genus include those of the New World species key by Lattke (2011); Sarnat and Economo (2012), Fiji species key; Zhou et al. (2012), China, Guangxi species key; Bharti and Wachkoo (2013), Indian species key; Rakotonirina and Fisher (2014), Malagasy species revision; Schmidt and Shattuck (2014), diagnosis and synoptic description of genus; Xu and He (2015), Oriental species; Zyranin (2016), Vietnam species key; Arimoto (2017), *Leptogenys modiglianii* species group revision; Sharaf et al. (2017), Arabian Peninsula species and a revised key to New World workers of *Leptogenys* by López-Muñoz et al. (2018). We here describe and illustrate a new species of the genus *Leptogenys* from India with a revised key to the known species of *chinensis*-group in Oriental Region.

## Materials and methods

Morphological observations were conducted using an Olympus SZX16 stereo zoom microscope. For digital images, a ProgRes0 CapturePro v.2.8.0. evolution digital camera was used on the same microscope with Combine ZP-Montage software. Later, images were cleaned with Adobe Photoshop CS6. Morphological terminology for measurements (given in millimetres) follows Lattke (2011), Bharti and Wachkoo (2013), Xu and He (2015) and include: HL; Maximum length of head capsule from anterior clypeal margin to mid-point of posterior head margin in full-face view. HW; Maximum width of head in full-face view. ML; Straight-line length of a mandible in full-face view, measured from the base at the insertion into the head capsule, to the apex. EL; Maximum length of eye as measured in oblique view of the head to show full surface of eye. SL; Maximum length of the scape excluding the basal neck and condyle. PW; Maximum width of pronotum in dorsal view. WL; Weber’s length of mesosoma, measured in lateral view from the anterior surface of the pronotum (excluding the collar) to the posterior margin of the propodeal lobes. PL; Maximum length of petiole from anterior process to posterior-most point of tergite, where it articulates with helcium. PDW; The maximum width of the petiole in dorsal view. PH; Height of the petiole measured in lateral view from the apex of the ventral (subpetiolar) process vertically to a line intersecting the dorsal-most point of the node (as defined in Xu and He (2015)). CI; Cephalic index: HW/HL × 100. MI; Mandibular index: ML/HW × 100. OI; Ocular index: EL/HW × 100. SI; Scape index: SL/HW × 100. LPI; Lateral petiole index: PH/PL × 100. DPI; Dorsal petiole index: PDW/PL × 100.

All specimens are deposited in Kashmir University Insect Collection, University of Kashmir, Srinagar (KUIC). One paratype will be deposited at Punjabi University Ant Collection, Punjab, India (PUAC) and one at California Academy of Sciences Collection, San Francisco, USA (CASC).

## Taxon treatments

### Leptogenys bhartii

Wachkoo, Maqbool, Akbar & Sharaf, 2018 sp. n.

urn:lsid:zoobank.org:act:3F871707-8D7E-4D18-85D3-6EADFCF3C8CB

#### Materials

- **Type status:** Holotype. **Occurrence:** recordedBy: Aijaz A Wachkoo; individualCount: 1; sex: worker; **Location:** country: India; stateProvince: Jammu & Kashmir; locality: Rajouri District, Thanamandi; verbatimElevation: 1600 m; verbatimCoordinates: 33.5379°N 74.3698°E; **Event:** samplingProtocol: hand collecting; eventDate: 08/11/2016; **Record Level:** institutionCode: KUIC
- **Type status:** Paratype. **Occurrence:** recordedBy: Aijaz A Wachkoo; individualCount: 4; sex: worker; **Location:** country: India; stateProvince: Jammu & Kashmir; locality: Rajouri District, Thanamandi; verbatimElevation: 1600 m; verbatimCoordinates: 33.5379°N 74.3698°E; **Event:** samplingProtocol: hand collecting; eventDate: 08/11/2016; **Record Level:** institutionCode: KUIC

#### Description

**Worker (Fig. 1)**

Morphometric data (Holotype in brackets): HL (1.79)-1.81, HW (0.92)-0.94, ML (0.91)-0.92, EL (0.45)-0.46, SL (2.22)-2.24, PW (1.06)-1.09, WL (3.22)-3.26, PL (1.13)-1.15, PDW (0.56)-0.58, PH (0.85)-0.87. Indices: CI (51)-52, MI 97-(98), OI (48)-49, SI 238-(241), LPI (75)-76, DPI (50)-51 (n=5).

Head trapezoidal in full-face view, lateral cephalic margin convex, posterior margin transverse; head distinctly wider anteriorly, converging posteriorly; eye large, weakly convex, placed just below the mid cephalic margin and within lateral margin; clypeus carinate with truncate apex, rugulose basally and smooth apically; anterolateral clypeal margin evenly converging medially, bending at rounded angle, with two median setae; scape surpasses posterior margin of head by about one-half its length; third antennal segment slightly more than 2× the length of second segment and about one-third longer than fourth segment; frontal groove deep, just extending to middle of eye level; mandible elongate, slender, external and basal margins parallel; basal tooth noticeable; basal sulcus distinct; masticatory margin edentate.

Mesosoma with promesonotal dorsal margin convex in lateral view; deeply impressed metanotal groove; metanotal and propodeal dorsal margin weakly convex, declivitous margin oblique; declivitous and dorsal propodeal margins meet through blunt obtuse angle in lateral view; mesometapleural suture well impressed with cross ribs; metapleural-propodeal suture barely impressed; propodeal spiracle oval, facing posterad; brief sulcus extends from spiracle to bulla. Mesonotum distinctly wider than long in dorsal view, propodeal declivitous face concave.

Petiole trapezoidal in lateral view, anterodorsal margin convex, node highest posterad with bluntly rounded apex, posterior margin leaning, with strong convexity basad; node triangular in dorsal view, much longer than broad, lateral margin weakly concave, anterior margin convex, posterior margin concave; subpetiolar process trapezoidal, angled posteriorly in lateral view. Gaster cylindrical, curved posteriorly, dorsal convex; cinctus between gastral segments prominent.

Body polished smooth and shiny with green metallic lustre, covered with sparse piligerous punctulae. Clypeus weakly rugulose posterolaterally; mandible shiny, punctate with superficial striation.

Body with abundant suberect hairs, denser on gaster, no appressed pubescence; pilosity mixed with short and long hairs.

Colour black for body, appendages brownish.

#### Diagnosis

The group is represented by eight valid species from the Oriental region (*L. assamensis* Forel, 1900, *L. chinensis* (Mayr, 1870), *L. confucii* Forel, 1912, *L. kraepelini* Forel, 1905, *L. laeviterga* Zhou, Chen, Chen, Zhou, Ban & Huang, 2012, *L. pangui* Xu, 2000, *L. peuqueti* (André, 1887) and *L. sunzii* Xu & He, 2015). Amongst the known species of the group, the new species resembles *L. chinensis*, but can be readily separated by the smooth medially converging anterolateral clypeal margins; propodeal declivity smooth and shiny, without transverse striations. Relatively narrower petiolar node in dorsal view, about twice as long as broad. Whilst in the latter, the anterior clypeal margin is distinctly laterally sinusoid; propodeal declivity transversely striate, not smooth and relatively broader petiolar node in dorsal view, about 1.3 times as long as broad. It also resembles *L. kraepelini* from which it can be distinguished by the following characters: gena smooth and shiny, without longitudinal rugae and third antennal segment slightly more than 2× the length of second segment whilst in the latter, gena are longitudinally rugulose and opaque and third antennal segment distinctly less than 2× the length of second segment.

#### Etymology

This species is named in honour of Dr. Himender Bharti for his outstanding contribution to the Indian ants.

#### Ecology

The specimens of this species were handpicked by dislodging a stone in a pine forest area from Thanamandi region of Rajouri district, located on the southerly foothills of the Pir Panjal Himalaya in the Indian State of Jammu and Kashmir. The region represents a transition zone between the subtropical Jammu and the temperate Kashmir provinces. The climate is broadly subtropical, although the foothill areas along the Pir Panjal range show dry temperate conditions, while subalpine and alpine conditions prevail higher up in the mountains. The vegetation mainly consists of Chir-pine (*Pinus roxburghii*) forests, broad-leaved deciduous forests, broadleaved evergreen forests and scrub forests, interspersed with frequent grassland patches and agricultural croplands.

## Identification Keys

### Keys to species of *Leptogenys chinensis*-group in Oriental Region (modified after Xu & He 2015)

**Table:** 

1	Petiolar node strongly elongate in lateral view, about 1.5 times as long as high.	2
–	Petiolar node moderately to weakly elongate in lateral view, less than 1.2 times as long as high.	3
2	Antennal scape relatively shorter, surpassing posterior head corner by about two fifths of its length. Petiolar node elongate trapezoidal in lateral view, with very short but distinct anterior margin. Body colour reddish-brown. Relatively smaller species with total length 6.5-7.0 mm.	***L. assamensis***
–	Antennal scape very long, surpassing posterior head corner by half of its length. Petiolar node triangular in lateral view, without anterior margin. Body colour black. Large species with total length 13.0-13.5 mm.	***L. pangui***
3	Petiolar node moderately elongate, as long as high or distinctly longer than high in lateral view.	4
–	Petiolar node weakly elongate, distinctly higher than long in lateral view, about 1.3-1.4 times as high as long.	8
4	Clypeus truncated at apex. Larger species with total length 8-11 mm.	5
–	Clypeus convex at apex. Smaller species with total length 4.5-7.0 mm.	7
5	Anterior clypeal margin distinctly laterally sinusoid; propodeal declivity transversely striate.	***L. chinensis***
–	Anterolateral clypeal margin, smooth; propodeal declivity smooth and shiny, without transverse striations.	6
6	Gena smooth and shiny, without longitudinal rugae and third antennal segment slightly more than 2× the length of second segment.	***L. bhartii* sp. n.**
–	Gena longitudinally rugulose and opaque and third antennal segment distinctly less than 2× the length of second segment.	***L. kraepelini***
7	Petiolar node distinctly longer than high in lateral view. Sides of mesothorax, metathorax and propodeum mostly smooth and shiny. Body colour black. Relatively larger species with total length 5.9-6.3 mm.	***L. peuqueti***
–	Petiolar node as high as long in lateral view. Sides of mesothorax, metathorax and propodeum mostly irregularly rugose and opaque. Body colour black, gaster blackish-brown. Relatively smaller species with total length 4.5 mm.	***L. confucii***
8	Clypeus truncated at apex. Eyes relatively smaller and occupying one fourth of head side. Petiolar node relatively longer in lateral view, about 1.3 times as high as long, dorsal margin distinctly longer than anterior margin, anterodorsal corner a rounded obtuse angle, the node obviously longer than broad in dorsal view.	***L. laeviterga***
–	Clypeus pointed and strongly convex at apex. Eyes larger and occupying one third of head side. Petiolar node relatively higher in lateral view, about 1.4 times as high as long, dorsal margin as long as anterior margin, anterodorsal corner a rounded right angle, the node as broad as long in dorsal view.	***L. sunzii***

## Supplementary Material

XML Treatment for Leptogenys bhartii

## Figures and Tables

**Figure 1a.**
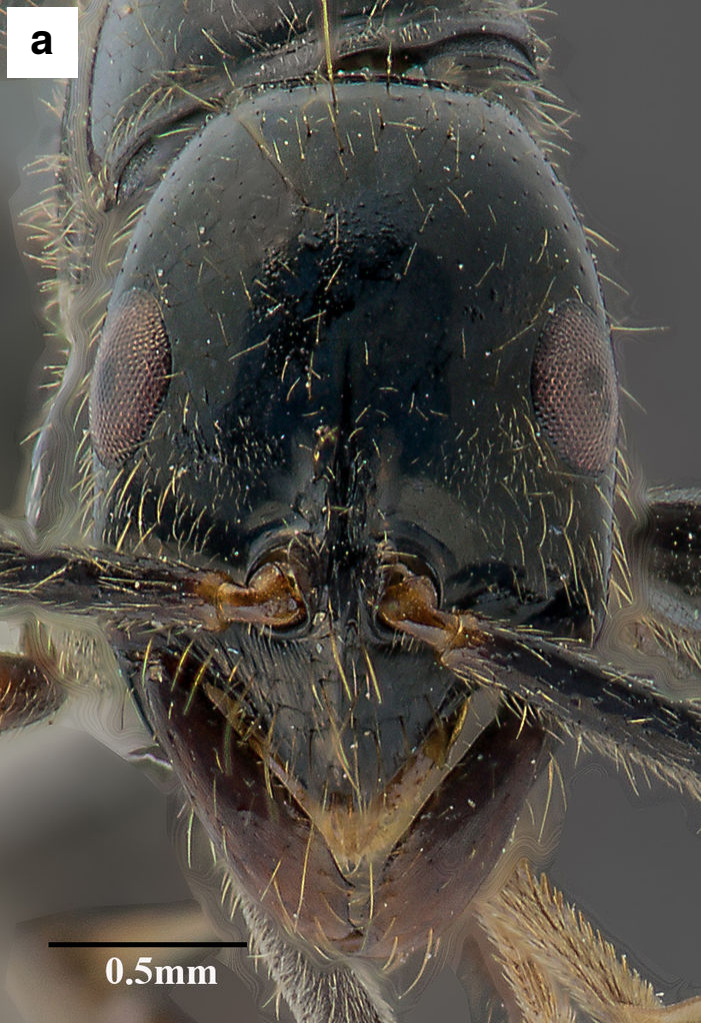
Head, full-face view

**Figure 1b.**
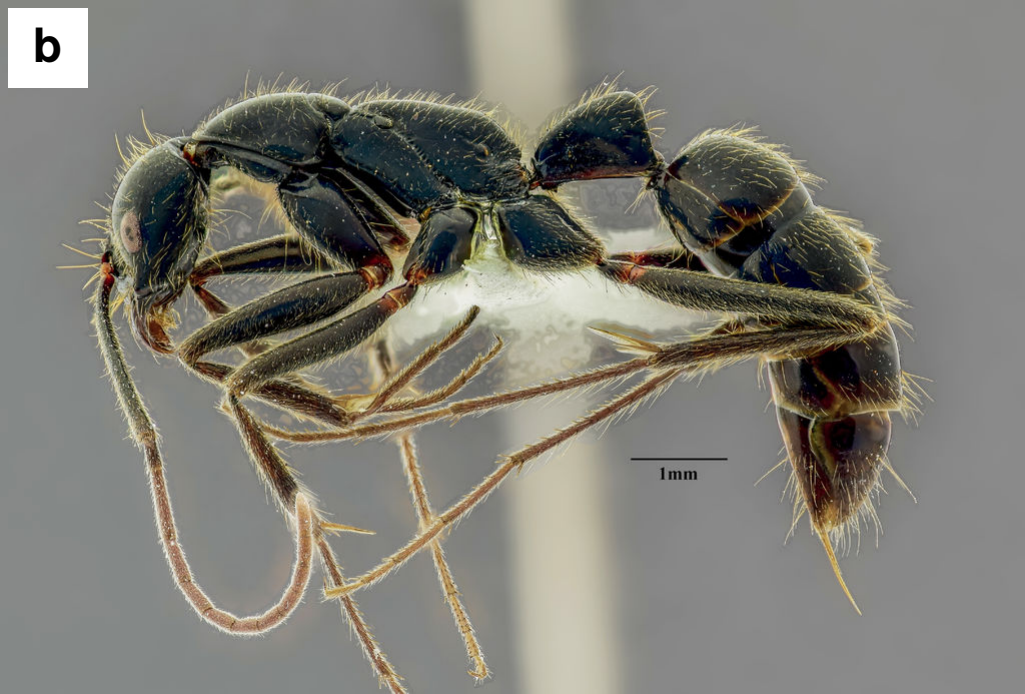
Body, lateral view

**Figure 1c.**
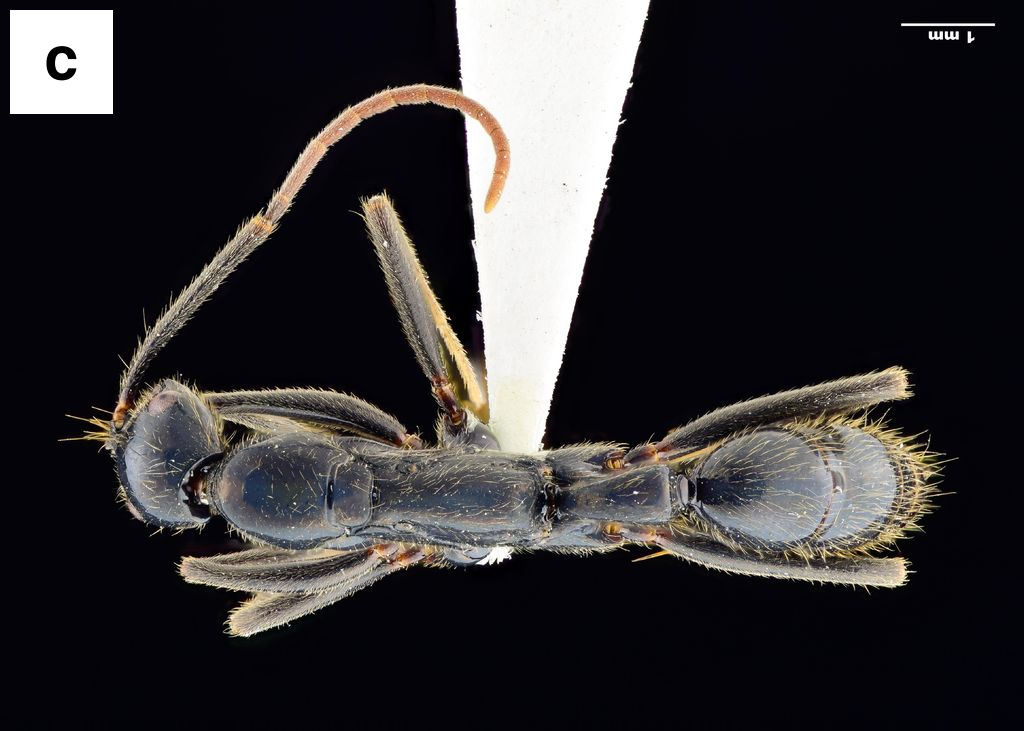
Body, dorsal view
